# Interpolated testing and content pretesting as interventions to reduce task-unrelated thoughts during a video lecture

**DOI:** 10.1186/s41235-022-00372-y

**Published:** 2022-03-26

**Authors:** Matthew S. Welhaf, Natalie E. Phillips, Bridget A. Smeekens, Akira Miyake, Michael J. Kane

**Affiliations:** 1 Department of Psychology, University of North Carolina at Greensboro, P.O. Box 26170, Greensboro, NC 27402-6170 USA; 2 Department of Psychology and Neuroscience, University of Colorado Boulder, Boulder, CO USA

**Keywords:** Mind wandering, Attention, Education, Testing, Pretesting

## Abstract

Considerable research has examined the prevalence and apparent consequences of task-unrelated thoughts (TUTs) in both laboratory and authentic educational settings. Few studies, however, have explored methods to reduce TUTs during learning; those few studies tested small samples or used unvalidated TUT assessments. The present experimental study attempted to conceptually replicate or extend previous findings of interpolated testing and pretesting effects on TUT and learning. In a study of 195 U.S. undergraduates, we investigated whether interpolated testing (compared to interpolated restudy) and pretesting on lecture-relevant materials (compared to pretesting on conceptually related but lecture-irrelevant materials) would reduce TUTs during a video lecture on introductory statistics. Subjects completed either a content-matched or content-mismatched pretest on statistics concepts and then watched a narrated lecture slideshow. During the lecture, half of the sample completed interpolated tests on the lecture material and half completed interpolated restudy of that material. All subjects responded to unpredictably presented thought probes during the video to assess their immediately preceding thoughts, including TUTs. Following the lecture, students reported on their situational interest elicited by the lecture and then completed a posttest. Interpolated testing significantly reduced TUT rates during the lecture compared to restudying, conceptually replicating previous findings—but with a small effect size and no supporting Bayes-factor evidence. We found statistical evidence for neither an interpolated testing effect on learning, nor an effect of matched-content pretesting on TUT rates or learning. Interpolated testing might have limited utility to support students’ attention, but varying effect sizes across studies warrants further work.

## Background

Students often lose focus and fail to attend to material presented during class, on video recordings, or in textbooks. Given the prevalence of such task-unrelated thoughts (TUTs), and the potential costs of chronic inattention to academic success, the science of learning has begun focusing its attention on distraction and mind wandering (for reviews, see Immordino-Yang et al., 2012; Lang, 2020; Pachai et al., 2016; Smallwood et al., 2007; Szpunar, Moulton, et al., 2013).

Most studies on TUTs during learning rely on experience-sampling methods that randomly interrupt students during a scholastic activity to report on their immediately preceding thoughts, particularly on whether their thoughts were focused on the learning task. Considerable research—in both laboratory and authentic educational settings—has documented TUT rates’ association with comprehension and learning outcomes, with students reporting more TUTs also demonstrating poorer comprehension and learning (e.g., Hollis & Was, 2016; Kane et al., 2017; Lindquist & McLean, 2011; Loh et al., 2016; Varao-Sousa & Kingstone, 2015; Wammes, Seli, et al., 2016b). Empirical studies have also focused on identifying contextual and individual-difference predictors of TUTs during learning (e.g., Bixler & D’Mello, 2016; Forrin et al., 2021; Hollis & Was, 2016; Kane, Carruth, et al., 2021; Lindquist & McLean, 2011; Locke & Jensen, 1974; Pham & Wang, 2015; Ralph et al., 2017; Risko et al., 2011, 2013; Schoen, 1970; Wammes et al., 2016a).

Much less research has targeted methods by which educators might limit TUTs, but there are some promising leads. High-tech methods might someday be widely available to help teachers or learners catch mind wandering on the fly and interrupt it, by analyzing subtle student behaviors that betray off-task thought, such as eye movements (e.g., Faber et al., 2018; Mills et al., 2021) and electroencephalography (e.g., Dhindsa et al., 2019). Until then, however, several common and easily implementable pedagogical practices, along the lines of “small teaching” (Lang, 2021), might be helpful.

For example, limited experimental evidence suggests that encouraging notetaking (versus not permitted notetaking) reduced TUTs during a video lecture, at least for students with less prior knowledge in the topic (Kane et al., 2017); correlational evidence also indicates that students who better take notes during lectures report fewer TUTs (Kane et al., 2017; Lindquist & McLean, 2011). As well, students sitting toward the back of lecture halls report more TUTs than do those toward the front (Lindquist & McLean, 2011), even after statistically controlling for other academic traits and habits (Kane, Carruth, et al., 2021; but see Wammes et al., 2019); these correlational findings suggest that sitting closer to the instructor might reduce TUTs but experiments that randomly assign students to seats are needed to establish causality.

The primary goal of the present study was to assess whether two interventions that prototypically benefit memory—interpolated testing and pretesting—may also facilitate focused attention during learning. As described below, several small but promising studies suggest that (a) periodically testing students on material they’ve recently encountered during a lecture, or (b) pretesting them on material they are about to encounter, reduces their TUT rates substantially compared to control conditions. The present study crossed both these interventions using video-learning materials previously demonstrated to yield high TUT rates and to produce individual differences in TUT rates that predict learning from, and situational interest evoked by, the lecture (Kane et al., 2017).

## Effects of interpolated testing and pretesting on TUTs

Among the few experimental intervention studies, the best replicated findings are that testing students on lecture-relevant information, either before or periodically during the lecture, reduces TUTs. Testing and pretesting effects are typically explored and evident in subsequent memory for learned material (for reviews, see Adesope et al., 2017; Carpenter & Toftness, 2017; Kornell & Vaughn, 2016; Metcalfe, 2017; Pan & Rickard, 2018; Roediger & Butler, 2011), but findings of “test-potentiated learning” (Chan et al., 2018) indicate that testing previously learned material can also benefit the subsequent learning of new material (e.g., Pastötter & Bauml, 2014; Wissman et al., 2011). Moreover, several recent laboratory studies using video lectures have found that either *interpolated testing* (where subjects are periodically tested *during* the lecture on material they’ve recently encountered) or *pretesting* (where subjects are tested on material *before* they’ve encountered it) also subsequently reduce TUTs during the lecture (Jing et al., 2016; Pan et al., 2020; Szpunar, Khan, et al., 2013).

### Interpolated testing and TUTs

Two articles, each reporting two studies, have examined the impact of interpolated testing on TUTs and learning from a video lecture (Jing et al., 2016; Szpunar, Khan, et al., 2013). Their logic is that in-lecture testing might motivate students to better attend to subsequent study materials. The findings are mostly supportive, but with some inconsistencies and ambiguities.

The Szpunar, Khan, et al. (2013), study presented subjects with a 21-min video about statistics divided into four segments, with post-segment activities varying between groups (*n* = 16 in each). In Experiment 1, each segment was followed by either a six-item test of the segment material, or no test (two groups); in Experiment 2, each segment was followed by a six-item test, no test, or a presentation of six test items with their answers provided for restudy, which is a more typical and appropriate control for studies of testing benefits (three groups). TUTs were assessed differently in each experiment. Experiment 1 measured TUTs at the end of the lecture via a 1–7 rating scale about the extent of mind wandering; such retrospective ratings, however, are vulnerable to memory and aggregation errors, as well as response biases, that may reduce their validity compared to in-the-moment thought reports (Kane, Smeekens, et al., 2021). Experiment 2 measured TUTs in the moment, with an experience-sampling probe inserted into each of the four lecture segments that asked whether subjects were just mind wandering.

Interpolated-testing groups reported less off-task thinking than did controls in both experiments. Subjects in Experiment 1 rated their attention as significantly less off-task during the lecture in the interpolated-testing condition (*Mdn* = 4) than in the no-testing condition (*Mdn* = 5). Similarly, in Experiment 2, subjects reported TUTs at significantly fewer probes in the interpolated-testing condition (*M* = 19%) than in the no-test (*M* = 41%) and restudy (*M* = 39%) conditions (*d* = 1.05 for the testing vs. restudy comparison). Although these findings suggest that interpolated testing reduced TUTs, both studies also allowed notetaking during the lecture, and subjects in the interpolated-testing group took more notes than did those in the other groups. It is possible, then, that in-lecture testing only indirectly affected TUTs by encouraging notetaking (Kane et al., 2017; Lindquist & McLean, 2011).

A follow-up study by Jing et al. (2016) compared interpolated testing and restudy groups (*n* = 18 in each) in two experiments, both of which also allowed notetaking. Here, eight thought probes were presented during a 40-min video lecture on public health. Experiment 1 assessed TUTs with “yes/no” mind-wandering thought probes and did not find a significant TUT-rate difference between interpolated-testing and restudy groups (*M*s = 21% and 24%, respectively; *d* = 0.15), thus failing to replicate prior findings.

Experiment 2 from Jing et al. (2016) modified the yes/no probes to assess five thought types, including thoughts related to the lecture topic but not about the here-and-now of the lecture (i.e., lecture-*related* off-task thought, such as reflecting on something mentioned earlier), in addition to lecture-*unrelated* off-task thought and “zoning out” without thought content. Here, the interpolated-testing group reported significantly lower TUT rates (lecture-unrelated plus zoning out; *M* = 3%) than did the restudy group (*M* = 15%), with *d* = 0.90. Lecture-related off-task thoughts showed the opposite pattern, with interpolated-testing subjects reporting significantly higher rates (*M* = 20%) than restudy subjects (*M* = 10%). Moreover, rates of lecture-related off-task thought correlated positively with posttest scores, *r*(25) = 0.45. Although the small sample size urges caution regarding these individual-differences results, they are directionally consistent with those reported by Kane et al. (2017) in a larger sample, *r*(180) = 0.26. In-lecture testing may therefore discourage potentially harmful off-topic thoughts while boosting potentially helpful on-task and lecture-related thoughts.

Note, however, that the first experiment by Jing et al. (2016) did not replicate the effect of interpolated testing on TUTs, so its benefits may not be robust across methodological variations. Alternatively, perhaps the benefits of in-lecture testing are reasonably robust, but small sample sizes (*n*s = 16 or 18 per group) made these studies vulnerable to false-negative errors and inflated estimates of effect size (e.g., Perugini et al., 2014; Schäfer & Schwarz, 2019). Finally, Jing et al., (2016; Experiment 1) and both studies reported in Szpunar, Khan, et al. (2013), showed increased notetaking with testing, which makes it difficult to establish a potential causal chain from testing to TUTs from the published studies.

Why might interpolated testing reduce TUTs? Chan et al. (2018) presented four theoretical frameworks for explaining how interpolated testing might potentiate future learning. Here, we discuss two of these frameworks, the “Resource” and the “Metacognitive” accounts, as they have suggested a possible role for interpolated testing in reducing TUTs. Resource accounts argue that testing may increase the available cognitive resources necessary for future learning, specifically because testing may redirect attention to the learning task and away from mind wandering (Jing et al., 2016; Pastötter et al., 2011; Szpunar, Khan, et al., 2013). With fewer resources dedicated to TUTs following testing, more will be available for the encoding of target information. The resource view does not explain, however, why testing episodes should redirect attention to the lecture more strongly than restudy episodes should. Alternatively, metacognitive accounts suggest that testing enhances learning beyond restudy because only testing alerts learners that they have not yet mastered the material. By this view, as learners become aware of their underperformance, they may use this feedback to refocus attention and put more effort in to learning the material (Cho et al., 2017; Lee & Ahn, 2017).

### Pretesting and TUTs

Only one study (in two experiments) has examined whether pretesting on information before it is presented, rather than testing on information after it is presented, also reduces TUT reports (Pan et al., 2020). The logic behind this approach is that, like interpolated testing, pretesting might increase attention to, or curiosity about, lecture-relevant information (Bull & Dizney, 1973; Hannafin & Hughes, 1986; Peeck, 1970; Pressley et al., 1990), or it might provide feedback to students that they have much to learn about the topic and so should pay close attention to the upcoming material (Bjork et al., 2013; Finn & Tauber, 2015). As well, and in contrast to testing after material is presented, pretesting might help highlight for students what specific aspects of the upcoming material is most critical, thereby scaffolding their attention allocation to relevant topics during the lecture (e.g., Peeck, 1970; Sagaria & Di Vesta, 1978).

Subjects in the two Pan et al. (2020) experiments viewed a 26-min video lecture, without taking notes, on signal detection theory. Each of four lecture segments ended with a probe to rate (0–100) how focused subjects’ attention had been on that entire video segment (again, such broad, retrospective judgments are vulnerable to validity-threatening errors of memory and aggregation). In Experiment 1, subjects either took an eight-item pretest on the upcoming segment’s material or solved unrelated math problems before each segment (*n*s ≈ 50 per group). In Experiment 2, subjects either took a 32-item pretest before the video (prevideo-pretested), took an eight-item pretest before each video segment, or solved math problems before each segment (*n*s ≈ 50 per group).

In both experiments, subjects who were pretested before each segment reported significantly higher attention ratings than did non-pretested subjects (Experiment 1 *M*s = 67 and 59, respectively, with *d* = 0.39; Experiment 2 *M*s = 67 and 50, respectively, with *d* = 0.74). In Experiment 2, the prevideo-pretested subjects showed similarly high attention ratings to the segment-pretested subjects (*M* = 71; *d* = 0.91 for contrast with non-pretested controls). Pretesting lecture material, either all at once or before each segment, thus appeared to reduce attention failures during learning. But, as in one of the studies showing that interpolated testing reduced mind-wandering (Szpunar, Khan, et al., 2013), attention was assessed with a retrospective-report measure of questionable construct validity (Kane, Smeekens, et al., 2021).

## Goals and hypotheses

The present study examined two intriguing but understudied interventions—interpolated testing and pretesting—to foster sustained and focused attention during learning from video lectures. Specifically, in a 2 × 2 study design, we asked whether interpolated testing or matched-content pretesting of lecture material (or both) would reduce subjects’ TUT reports during learning from a narrated-slideshow lecture on introductory statistics, a context previously established to yield valid measurement of TUTs and learning (Kane et al., 2017). Before these promising interventions can be applied to actual educational settings, the field must better establish their robustness and effect sizes.

The present study addressed our concerns with prior studies noted earlier. For example, we addressed measurement concerns by assessing TUTs with validated thought probes of immediately preceding experience (that also allowed for the reporting of lecture-related as well as lecture-unrelated off-task thought; Jing et al., 2016; Kane et al., 2017). Like prior studies investigating the effect of interpolated testing on TUTs (Jing et al., 2016; Szpunar, Khan, et al., 2013), we contrasted an in-lecture testing group to a restudy control group; we did not, however, allow notetaking, which in turn allowed us to assess whether interpolated testing decreases TUTs directly, without possibly doing so indirectly by increasing notetaking.

The present study’s control condition for the pretesting effect also isolated a different potential mechanism for reducing TUTs from that proposed to drive any effects of testing (i.e., motivating sustained attention based on learning feedback, per the metacognitive account of test-potentiated learning). Unlike Pan et al. (2020), who, consistent with most of the pretesting literature, contrasted pretesting to no-pretesting groups, we compared a pretesting group to a control group that also took a pretest, but on statistics topics not covered in the lecture (i.e., mismatched content). Both pretests should similarly provide subjects with feedback that they have little knowledge about statistics and still have much to learn, and so both conditions should similarly engage metacognition and motivate sustained attention to the lecture. Only the matched-content pretest condition, however, highlighted for subjects the specific information from the lecture that would be most important for the final test, and so only the matched-content condition could scaffold attention to the most task-relevant material. Consistent with this possibility, some prior research has found that pretesting benefits for learning are found only for the specific topics that are pretested, rather than generalizing to related information in the learning material (e.g., Bull & Dizney, 1973; Pressley et al., 1990; Richland et al., 2009; Sagaria & Di Vesta, 1978; but see Carpenter & Toftness, 2017).

Our primary hypotheses were that: (a) subjects who completed in-lecture tests for the lecture material would show decreased rates of TUTs, and possibly increased rates of lecture-related off-task thought, compared to subjects who restudied the information at matching intervals; (b) subjects who completed a pretest on the upcoming lecture material (i.e., matched content) would report fewer TUTs, and possibly more instances of lecture-related off-task thought, than would subjects who completed a lecture-unrelated pretest (i.e., mismatched content).

As discussed above, if our study design elicited significant effects of both interpolated testing and matched-content pretesting on TUTs, it should do so via different mechanisms for each (learning feedback to facilitate metacognition from interpolated testing, versus highlighting key to-be-learned information from pretesting). Crossing these interventions, then, should most likely result in additive main effects. However, because over-additive effects of receiving both interventions were possible (although not specified by any prior testing or pretesting research), as a more exploratory exercise we also tested for an interaction of interpolated testing and pretesting content match on TUT rates.

Our secondary hypotheses concerned outcome measures beyond TUT rate. As in our previous study of TUTs during learning from videos (Kane et al., 2017), subjects completed a posttest on the lecture material and reported their situational interest in statistics elicited by the video. The testing effect and pretesting literatures, as well as the studies of interpolated testing and pretesting on TUTs (Jing et al., 2016; Pan et al., 2020; Szpunar, Khan, et al., 2013), suggest that both interpolated testing and pretesting should improve posttest performance in addition to reducing TUTs. Although one might expect that a side effect of decreasing TUTs would be to also increase situational interest in the learning material (Kane et al., 2017), the study by Jing et al. (2016) found no effect of interpolated testing on interest stimulated by the lecture; we therefore we did not have strong predictions for the potential effects of interpolated testing or pretesting on interest.

## Method

Below we report how we determined our sample size and all data exclusion decisions, experimental manipulations, and measures for this study (Simmons et al., 2012). Some materials and procedures were identical to those from our study on notetaking and TUTs during a video lecture (Kane et al., 2017). The study received ethics approval from the Institutional Review Board of the University of North Carolina at Greensboro (UNCG). All materials for the current study are available at the OSF site, https://osf.io/6ujsg/. Video lecture materials are available from the Kane et al. (2017) OSF site, https://osf.io/u5bnw/.

### Subjects and sample-size determination

We did not preregister a sample size based on power analyses, but we aimed to collect usable data from 200 subjects, yielding 100 subjects per group for each main effect (interpolated testing vs. restudy; matching vs. mismatching pretests). This sample size is five times as large as those in prior experiments on interpolated testing and TUTs (Jing et al., 2016; Szpunar, Khan, et al., 2013), about twice as large as those in prior experiments on pretesting and TUTs (Pan et al., 2020), and similar to our prior study of TUTs with these materials (Kane et al., 2017). As noted above, our primary hypotheses were for main effects of interpolated testing and matched-content pretesting; we expected additive effects for these interventions when combined in our 2 × 2 design, but over-additive interactions were possible and of applied interest.

**Fig. 1:**
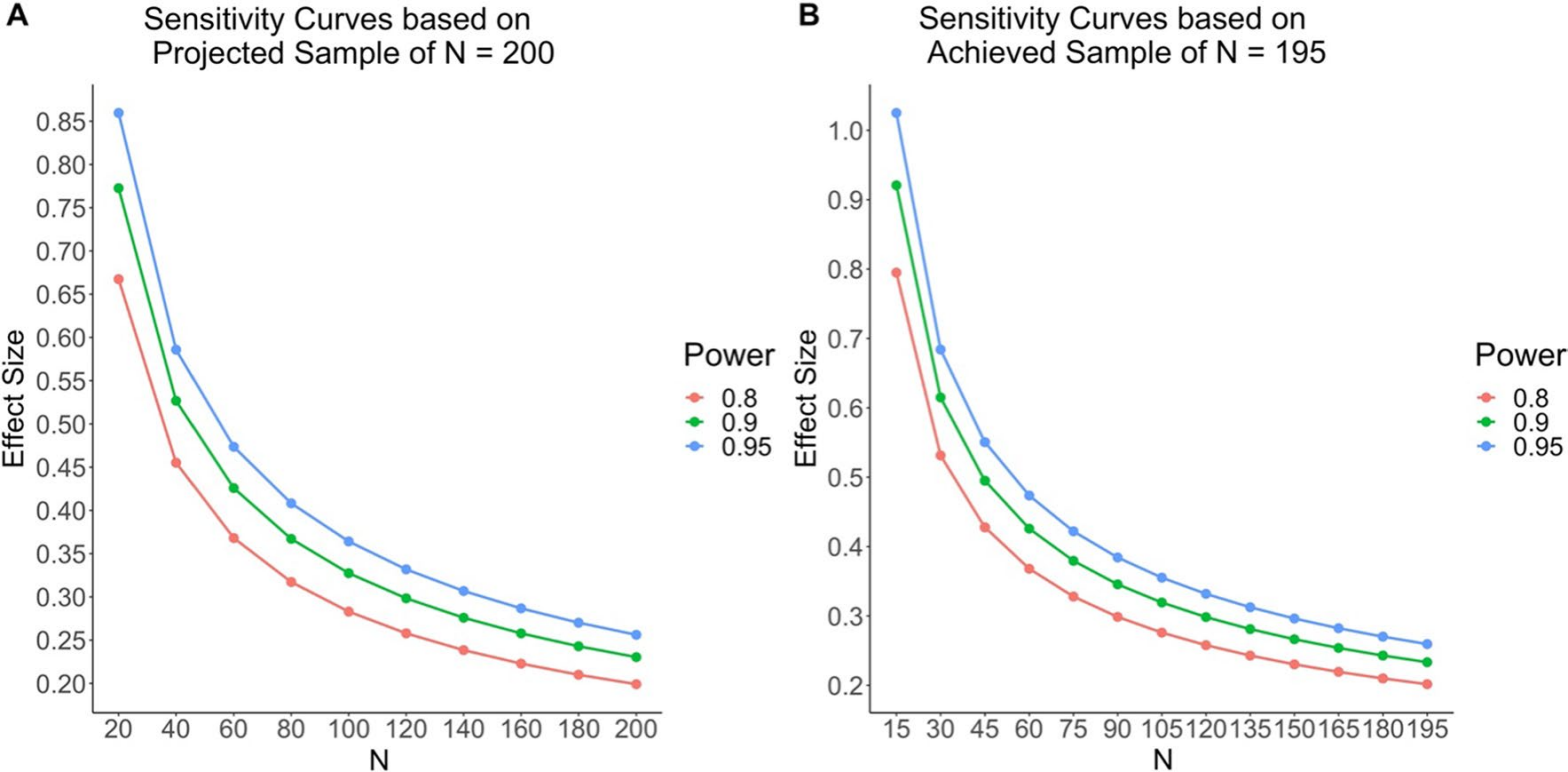
Sensitivity curves based on projected (Panel A) and achieved (Panel B) sample sizes. Effect sizes are Cohen’s *f*

We report sensitivity analyses for ANOVA main effects using G*Power (Faul et al., 2007) for 80%, 90% and 95% power (α = 0.05); the curves are displayed in Fig. 1 (panel A). With *N* = 200, we could detect an effect between *f* = 0.20–0.26 (with 80% and 95% power, respectively)—conventionally “medium-sized” effects (for comparison with effect sizes in the literature based on *t*-tests, Cohen’s *d* = [Cohen’s *f* × 2], assuming equal sample sizes). As noted in the Results section, our final sample after data exclusions was *N* = 195; the corresponding sensitivity analyses (see panel B) also indicated 80% and 95% power to detect main effects of *f* = 0.20 and 0.26, respectively.

For comparison, prior significant effect of testing on TUTs (Jing et al., 2016, Experiment 2; Szpunar, Khan, et al., 2013) yielded effect sizes in the range of *f* = 0.44–0.49 (but Jing et al., 2016, Experiment 1, found a null effect, *f* = 0.075). Likewise, the Pan et al. (2020) effects of pretesting (vs. no pretest) on TUTs yielded effect sizes of *f* = 0.20 (Experiment 1) and *f*s = 0.37 and 0.46 (Experiment 2, for interpolated and blocked pretests, respectively). With a sample size of 195, we would be able to detect an effect of roughly half the size of the significant interpolated-testing effects and of the smallest pretesting effects reported in previous studies. Therefore, our design was well powered for these main effects. Note, however, that any interaction effect of these variables would have to be unusually large to be detected, requiring only cautious conclusions about additivity.

We consented 277 undergraduates from UNCG, a comprehensive state university and minority-serving institution for African American students. We tested more subjects than our target sample size because, following Kane et al. (2017), we planned to drop data from subjects who indicated that they had previously taken a statistics course (see below). Eligible subjects were between the ages of 18–35 and participated for either partial credit toward an Introductory Psychology requirement or $25.00. We randomly assigned subjects to one of four conditions based on our 2 (Interpolated Activity: Testing vs. Restudy) × 2 (Pretest Content: Match vs. Mismatch) factorial design, with the constraint that all subjects within a session were assigned to the same condition.

Of our retained 195 subjects, 72% self-identified as female and 28% as male; mean age was 19.06 years (SD = 1.87). The self-reported racial breakdown of our final sample was 52% White (European or Middle Eastern descent), 37% Black (African or Caribbean descent), 7% Multiracial, 3% Asian, 1% Native Hawaiian or Pacific Islander, and 0% Native American or Alaskan Native (*n* = 2 missing). Finally, self-reported ethnicity, asked separately, was 6% Hispanic or Latino.

### Procedure, materials, and equipment

#### Computers, software, and peripheral equipment

Each subject completed the study on a Mac Mini linked to an Acer 22-in LCD monitor. Audio for the video lecture was presented via Koss UR-20 headphones. For the pretest and posttest, we provided subjects with a calculator (Sharp EL243SB). We programmed all measures and the video lecture in E-prime 2.0 (Psychology Software Tools, Pittsburgh, PA).

#### Overall procedure

Subjects completed the study individually or in groups of up to four. The experimenter remained in the testing room during the study and read aloud all on-screen instructions. Following the completion of a given task, subjects in group sessions waited until everyone finished before moving on to the next task. Most sessions lasted 90–120 min. Following informed consent, subjects completed the following measures and tasks in the order described.

#### Questionnaires, measures, and stimuli

##### Statistics background questionnaire

A single-item questionnaire asked subjects to report, by clicking on a box located next to their answer, if they had taken a formal course on statistics (Kane et al., 2017). The response options were: (A) no statistics courses taken; (B) college statistics course in Psychology on this campus; (C) college statistics course(s) in other Departments on this campus; (D) college statistics course(s) in other institutions/universities; (E) high school statistics course(s); (F) online statistics courses (e.g., Khan Academy, iTunes-U). Data from subjects reporting any statistics coursework (responses B–F) were dropped from analyses.

##### Statistics pretest

Depending on pretest-content condition, subjects next completed one of two 10-item multiple-choice pretests with the aid of a calculator and no time limit. Each question was followed by 6 or 7 answer choices labeled A–F or A–G with a checkbox next to each answer choice. Subjects recorded their answer by mouse-clicking the box next to their answer choice. Subjects also provided a confidence report for each item: (a) had to guess and had little confidence; (b) had to guess but were still somewhat confident; (c) knew the answer and/or were highly confident.^1^ The main dependent measure from the pretest, regardless of condition, was the proportion of 10 items answered correctly. Moreover, both pretests were designed such that subjects should answer few items correctly without having previously learned statistics.

Subjects in the matched-content pretest condition completed items that reflected the upcoming video-lecture content, and that were identical to those to be presented as Part 1 of the posttest (as in Kane et al., 2017, subjects were unaware that they would be tested on these same items after the lecture). Subjects in the mismatched-content pretest condition completed a set of items that were relevant to introductory statistics courses (and were inspired by several introductory statistics textbooks), but these topics were not covered in the upcoming video lecture and did not appear in the posttest.

##### Video lecture

We adapted the 52-min video lecture used by Kane et al. (2017), which was a narrated PowerPoint presentation showing text and images that introduced basic statistical concepts (e.g., samples, populations, frequency distributions, central tendency), taught the steps to calculate the standard deviation of a set of scores, and demonstrated the utility of the mean and standard deviation in interpreting one’s own SAT scores. This video consisted of 31 segments, the first of which lasted for 5 min, and the remaining 30 segments were between 1:08 and 1:51 min in length. The segments were organized in 5 blocks, each of which ended with either a set of interpolated-test or interpolated-restudy items (a between-subject manipulation).

Each interpolated break presented six items: three multiple-choice questions with four response options each (e.g., *If you knew a sample’s standard deviation, how do you calculate its variance? a) take the square root of the number; b) square the number; c) divide it by N; d) add it to the sum of* squares), and three short-answer questions (e.g., *How would the median of the following sample of scores: 3,4,7,8,9 change if the largest value (9) changed to 49?*). Subjects saw one item at a time for 20 s and either answered the question (in the testing condition) or studied the highlighted (italicized and underlined) answer (in the restudy condition) within that time. After 20 s, the next item appeared onscreen (89% of items were answered within 20 s; unanswered items were scored as incorrect). The lecture video resumed after completion of the final item. The interpolated items were related to the content of the immediately preceding lecture block, but they did not match any of the pretest or posttest items. Subjects in the interpolated-testing conditions received no accuracy feedback.

##### Video-embedded thought probes and instructions

Before beginning the video, we instructed subjects about the periodic thought probes that would appear throughout the lecture (see Kane et al., 2017, for more details about instructions). Each probe presented a green screen with 7 response options listed, for subjects to report the content of their immediately preceding thoughts. These thought-report options appeared, and were explained, as follows (only the numbers and italicized labels here appeared on each probe screen):

1. *On-task on the lecture*: Thoughts about the in-the-moment video-lecture content
2. *Lecture-related ideas*: Thoughts about some aspect of the lecture topic, but not what was currently happening in the video
3. *How well I’m understanding the lecture*: Evaluative thoughts about comprehending (or not) the lecture material
4. *Everyday personal concerns*: Thoughts about normal everyday things, life concerns, or personal worries
5. *Daydreams*: Fantasies or unrealistic thoughts
6. *Current state of being*: Thoughts about one’s current physical or mental state (e.g., sleepy, hungry, or fascinated)
7. *Other*: Any thoughts not fitting into the other categories.

During the video, subjects saw 15 probes. As in Kane et al. (2017), probes were presented between video segments with the constraint that probes could not appear after three consecutive video segments. We also incorporated an additional constraint that probes could not appear at the end of a block immediately preceding an interpolated test or restudy break. (Note that Kane et al. presented 20 probes, but here we replaced one probe per block with the interpolated activity.) We scored thought reports as follows (consistent with Kane et al., 2017): TUTs were defined as the proportion of thought reports with responses 4–7, lecture-related off-task thoughts were the proportion of reports with response 2, and comprehension-related off-task thoughts were the proportion of reports with response 3.

##### Situational interest questionnaire

As in Kane et al., (2017; modified from Linnenbrink-Garcia et al., 2010), the video lecture was immediately followed by 10 items assessing interest in the video and in statistics (e.g., “*I found the content of this video lecture personally meaningful*,” “*To be honest, I just don’t find statistics interesting*”). Subjects rated each item on 5-point scale with the following options: (1) strongly disagree, (2) somewhat disagree, (3) neither agree nor disagree, (4) somewhat agree, and (5) strongly agree. The dependent measure was the average score of all items, after reverse scoring appropriate items. Although the main analyses in Kane et al. (2017) excluded the three items about interest in the field of statistics (as opposed to interest in the lecture, itself), those items behaved similarly to the rest of the scale, so we included all 10 items here.

As in Kane et al. (2017), the retention interval between the video-lecture and posttest was fixed by presenting each questionnaire item onscreen for 9.5 s. For the first 4.5 s, the item appeared against a white screen. For the final 5 s, the screen turned yellow to indicate that subjects should now type their numerical response. Regardless of when subjects responded, each item stayed onscreen for the full 9.5 s. The questionnaire included one attention-check item with the same response scale (“I saw this exact stats video lecture in my preschool art class.”). Data from subjects who responded to this item with *neither agree nor disagree, somewhat agree,* or *strongly agree* were removed from analyses of situational interest (*n* = 14).

##### Statistics posttest

We used the same three-part, untimed posttest as Kane et al. (2017). Specifically, Part 1 included 10 multiple-choice questions (the same as those appearing in the matched-content pretest); Part 2 required subjects to calculate the standard deviation of a set of four numbers; Part 3 required subjects to calculate the standard deviation of a new set of five numbers, but each of five calculation steps was labeled and completed in turn (i.e., first calculate the mean, then the deviation scores, then the sum of squares, then the variance, and then the standard deviation).

For Part 1, subjects mouse-clicked on their answer on-screen, just as in the pretest. For Parts 2 and 3, subjects were provided with a packet to complete their calculations, with the aid of a calculator; for Part 2, subjects used one sheet of packet paper, and for Part 3, each of the five calculation steps was labeled and completed in a separate sheet of paper. Subjects completed their work on paper first and then typed in their answer on the computer and pressed ENTER to record it. As in Kane et al. (2017), the dependent measure for the posttest was calculated as the mean score across the three parts after z-scoring the raw score for each part across whole sample (partial credit was granted in Parts 2 and 3, as in Kane et al., 2017).

##### Demographic questionnaire

Subjects completed a demographics questionnaire at the end of the session, reporting on their self-identified Sex/Gender (open-ended), age (open-ended), ethnicity (Hispanic or Latino vs. not Hispanic or Latino), race (Asian; Black: African or Caribbean descent; Native American or Alaskan Native; Native Hawaiian or Pacific Islander; Multiracial; White: European or Middle Eastern descent), and university major (open-ended; unanalyzed).

## Results

All data aggregation and analyses were performed in R (R core team, 2020) using *tidyverse* (Wickham, 2019). ANOVAs and calculation of effect sizes were performed in the *afex* (Singmann et al., 2020), and *effectsize* (Ben-Shachar et al., 2020) packages; data visualizations were created using *ggplot2* (Wickham, 2016). Data and analysis scripts are available at the OSF site, https://osf.io/6ujsg/

### Data analysis plan

We adopted a .05 α level for null hypothesis significance testing inferences from our 2 × 2 ANOVAs and report 95% confidence intervals where applicable. For experimental comparisons of interest (e.g., interpolated testing versus restudy), we also conducted *t*-tests with corresponding Bayes Factors (BFs) to compare predictive performance of competing models with a continuous measure of evidence (Kass & Raftery, 1995). Null models reflected a Cauchy distribution centered around 0 with a scaling parameter of 0.707. This corresponds to a probability that 50% of the distribution was between *d* =  − 0.707 and 0.707 (Rouder et al., 2009). Given the combination of small sample sizes and mixed effect sizes in the prior testing–TUT literature (with some very large effects and one very small effect), this is a reasonable expectation of effect size (Schmalz et al., 2021). BFs were calculated using the *BayesFactor* package (Morey & Rouder, 2018). We interpreted BF_10_ < 0.33 (1/3) as providing modest evidence for the null relative to the alternative hypothesis and BF_10_ > 3.0 as providing modest evidence for the alternative hypothesis relative to the null, and BF_10_ < 0.10 (1/10) as providing strong evidence for the null relative to the alternative hypothesis and BF_10_ > 10 providing strong evidence for the alternative hypothesis relative to the null.

### Data loss

We based initial data-exclusion decisions on experimenter session notes while blinded to subjects’ performance, thought-report, and questionnaire data. We dropped data from two subjects for falling asleep multiple times, from six subjects for leaving the session early, from three subjects who were assigned to the wrong condition in the session, and from four subjects who were in a session that was significantly delayed and disrupted by one subject (total dropped = 15). Additionally, as in Kane et al. (2017), we dropped data from 66 subjects who reported they had previously completed a statistics course. Although Kane et al. (2017) also dropped data from subjects scoring ≥ 60% on the pretest, the only two subjects who did so here were already dropped for having completed a statistics course. Finally, we dropped data from one subject who reported an age that was outside our eligibility range of 18–35 years. The final sample consisted of 195 subjects (as noted above, we additionally dropped situational interest data from 14 subjects who failed an attention check embedded in the questionnaire).

### Preliminary analyses of pretest performance

**Fig. 2:**
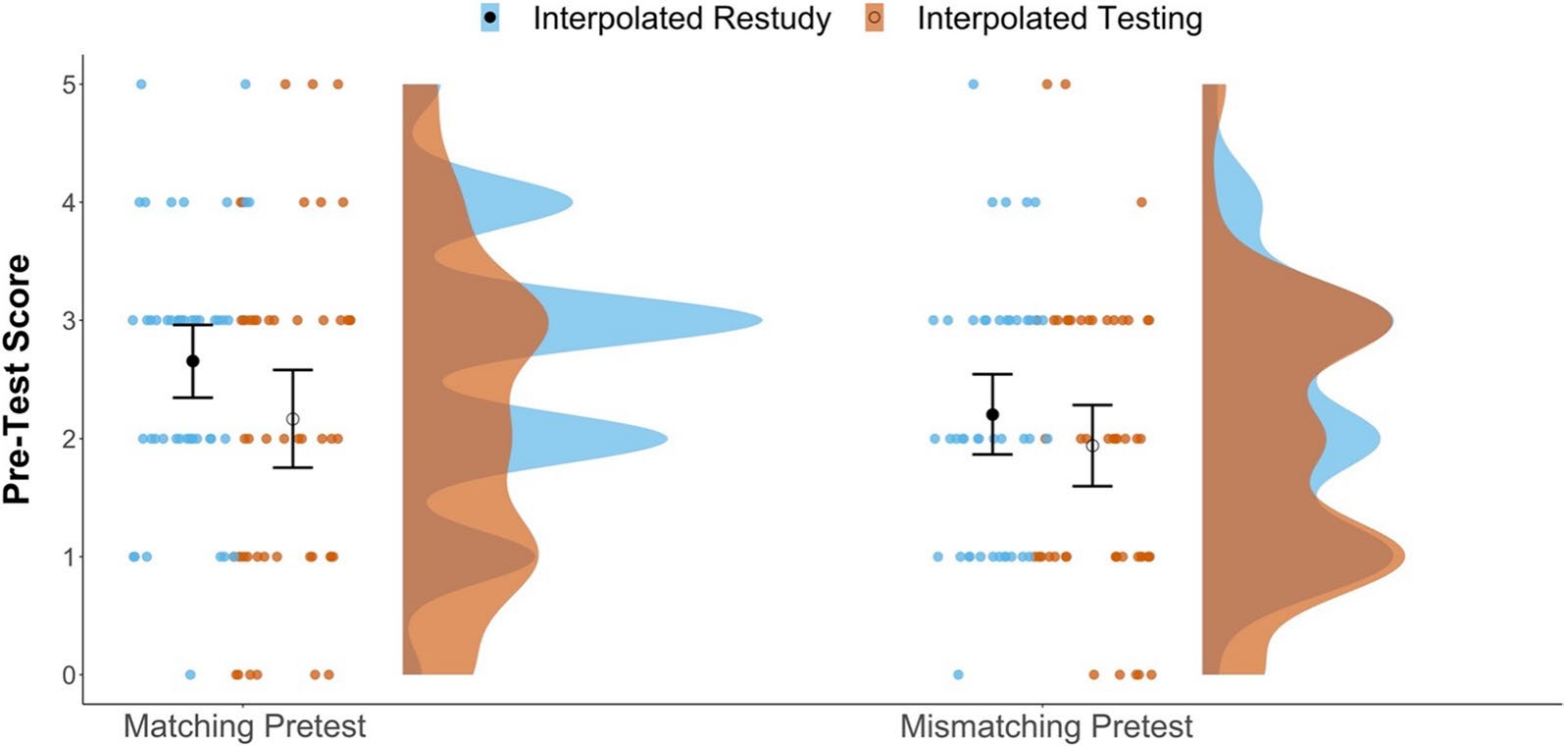
Raincloud plots depicting differences in pretest scores between conditions. Dots represent individual subject means in each condition. The closed black dots represent group-level mean estimates for the Restudy conditions; open circles represent the group-level mean estimates for the Testing conditions. Error bars are 95% confidence intervals

Table 1 presents descriptive statistics for all variables of interest, by interpolated activity (testing vs. restudy) and pretest content (matching vs. mismatching). Before assessing whether TUT rates or posttest performance benefitted from either intervention, we tested whether pretest scores suggested any preintervention group differences, despite randomization to conditions. Pretest scores for the four experimental conditions are shown in Fig. 2.

**Table 1:** Descriptive statistics by pretest (content-match vs. mismatch) and interpolated activity (testing vs. restudy) conditions

Dependent variable	Experimental conditions															
	Matched testing (*n* = 48)				Matched restudy (*n* = 52)				Mismatched testing (*n* = 51)				Mismatched restudy (*n* = 44)			
	M	SD	Min	Max	M	SD	Min	Max	M	SD	Min	Max	M	SD	Min	Max
Pretest	2.17	1.42	0.00	5.00	2.65	1.10	0.00	5.00	1.94	1.22	0.00	5.00	2.20	1.11	0.00	5.00
TUT Rate	0.38	0.25	0.00	0.87	0.47	0.24	0.00	1.00	0.42	0.25	0.00	1.00	0.48	0.26	0.00	1.00
Lecture-Related	0.15	0.11	0.00	0.47	0.14	0.12	0.00	0.53	0.15	0.14	0.00	0.53	0.17	0.11	0.00	0.40
Comp-Related	0.14	0.14	0.00	0.60	0.12	0.13	0.00	0.47	0.15	0.13	0.00	0.47	0.15	0.14	0.00	0.53
Posttest Part 1	4.29	2.09	0.00	8.00	4.37	2.39	0.00	9.00	4.59	2.29	1.00	10.00	4.57	2.06	1.00	8.00
Posttest Part 2	2.17	1.71	0.00	5.00	2.16	1.63	0.00	5.00	2.25	1.57	0.00	5.00	2.35	1.70	0.00	5.00
Posttest Part 3	2.47	1.68	0.00	5.00	2.75	1.65	0.00	5.00	2.83	1.80	0.00	5.00	3.06	1.84	0.00	5.00
Posttest Total	−0.10	0.78	−1.68	1.25	−0.03	0.89	−1.52	1.70	0.04	0.87	−1.52	1.70	0.11	0.81	−1.37	1.40
Sit. Interest	2.74	0.61	1.50	3.90	2.75	0.64	1.60	4.00	2.63	0.78	1.00	4.30	2.89	0.80	0.90	4.44

Matched = content-matched pretest; Mismatched = content-mismatched pretest; Pretest = number correct pretest items; TUT Rate = proportion of thought reports indicating task-unrelated thoughts; Lecture-Related = proportion lecture-related off-task thoughts; Comp-Related = proportion comprehension-related off-task thoughts. Posttest Parts 1–3 = number correct posttest items per part; Posttest Total = z-score average across all posttest parts; Sit. Interest = situational interest scale score. *N*s for Situational Interest outcome: Matched Testing = 44; Matched Restudy = 49; Mismatched Testing = 47; Mismatched Restudy = 41

The results of the 2 (Pretest Content: Match vs. Mismatch) × 2 (Interpolated Activity: Testing vs. Restudy) ANOVA on pretest performance indicated neither a significant main effect of pretest-content match, *F*(1, 191) = 3.71, *p* = 0.056, *η*_*p*_^2^ = 0.019, nor a significant interaction with interpolated activity, *F*(1, 191) = 0.41, *p* = 0.524, *η*_*p*_^2^ = 0.002. We find no evidence, then, that the two content-matched versus mismatched pretests differed in difficulty (*M*s = 2.42 and 2.06, respectively). Unexpectedly, however, the ANOVA indicated an effect of interpolated activity, with subjects who would subsequently restudy at interpolation breaks scoring significantly higher on the pretest (*M* = 2.45) than did subjects who would subsequently be tested at interpolation breaks (*M* = 2.05), *F*(1, 191) = 4.59, *p* = 0.033, *η*_*p*_^2^ = 0.023.

As noted earlier, to further explore all main effects of interest, we conducted follow-up *t*-tests to provide corresponding Bayes Factors (BF) and Cohen’s *d* indicators of effect size. Table 2 presents these results for all key experimental contrasts in the study. The BF for the significant effect of interpolated activity here indicated only weak evidence that the data were more likely under the alternative than the null hypothesis.

**Table 2:** Follow-up *t*-tests, Cohen’s *d*, and Bayes Factors (BF_10_) for Primary Dependent Variables in Testing Versus Restudy Conditions and Content-Matched Versus Content-Mismatched Pretest Conditions

Dependent variables	Experimental comparisons					
	Testing vs. restudy			Matched vs. mismatched pretest		
	*t*-test	*d* [95% CI]	BF_10_	*t*-test	*d* [95% CI]	BF_10_
Pretest	*t*(193) = − 2.26*	− 0.32 [− 0.61, − 0.04]	1.67	*t*(193) = − 2.03*	− 0.29 [− 0.57, − 0.01]	1.05
TUT Rate	*t*(193) = − 2.00*	− 0.29 [− 0.57, − 0.00]	0.99	*t*(193) = 0.52	0.08 [− 0.21, 0.37]	0.18
Lecture-Related	*t*(193) = − 0.26	− 0.04 [− 0.32, 0.24]	0.16	*t*(193) = 0.67	0.10 [− 0.19, 0.38]	0.19
Comp-Related	*t*(193) = 0.73	0.11 [− 0.18, 0.39]	0.20	*t*(193) = 1.13	0.16 [− 0.12, 0.44]	0.28
Posttest Total	*t*(193) = − 0.47	− 0.07 [− 0.35, 0.21]	0.17	*t*(193) = 1.08	0.16 [− 0.13, 0.44]	0.27
Sit. Interest	*t*(179) = − 1.24	− 0.18 [− 0.48, 0.11]	0.33	*t*(179) = 0.06	0.01 [− 0.28, 0.30]	0.16

Matched = pretest content matched posttest; Mismatched = pretest content mismatched posttest; Pretest = number correct pretest items; TUT Rate = proportion of thought reports indicating task-unrelated thoughts; Lecture-Related = proportion lecture-related off-task thoughts; Comp-Related = proportion comprehension-related off-task thoughts; Posttest Total = z-score average across all parts; Sit. Interest = situational interest scale score

^*^ *p* < .05

Despite the weak effect, the statistically significant pretest findings suggest that we should analyze posttest performance, and all other outcomes of interest, both with and without including pretest score as a covariate. For these supplemental ANCOVAs, we standardized pretest scores within each pretesting condition, given that the content-matching and content-mismatching conditions presented different pretest items. All ANCOVA results are reported in Appendix A; in no case did the ANCOVA results yield different conclusions than did the ANOVAs without the pretest score covariate.

### Primary analyses of thought reports

Here, we analyze whether TUT rates, or other varieties of off-task thought, were affected by our experimental interventions—interpolated testing versus restudy, content-matched versus content-mismatched pretests, or both.

#### TUT rates

As seen in Table 1, subjects averaged reporting TUTs to about 40–50% of the probes during the video lecture, consistent with our prior study using the same video content and thought probes (Kane et al., 2017). Also consistent with prior findings, there was considerable individual variability in TUT rates, with standard deviations of about 25% around those means.

**Fig. 3:**
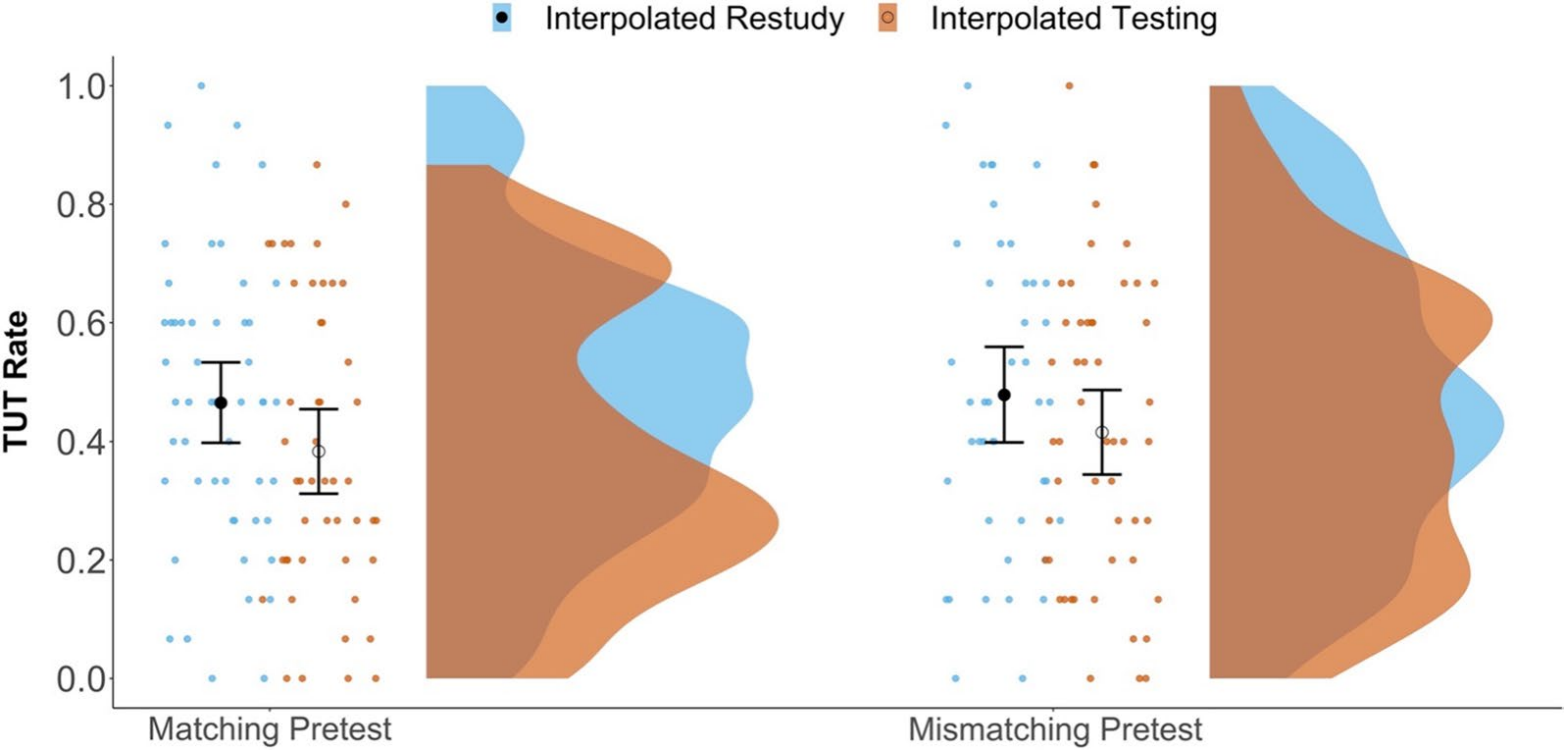
Raincloud plots depicting TUT rates (the proportions of thought reports indicating TUTs) in each condition. Dots represent individual subjects’ TUT rates. The closed black dots represent group-level mean estimates for the Restudy conditions; open circles represent the group-level mean estimates for the Testing conditions. Error bars are 95% confidence intervals

Our primary question focused on the potential effects of interpolated activity and pretest match on TUT rates. As suggested by Fig. 3, the 2 × 2 ANOVA indicated a just-significant main effect of interpolated activity, *F*(1, 191) = 4.05, *p* = 0.046, *η*_*p*_^2^ = 0.021, with lower TUT rates for subjects in the interpolated testing condition (*M* = 0.40) than in the restudy condition (*M* = 0.47). There was no significant effect of pretest-content match, *F*(1, 191) = 0.40, *p* = 0.526, *η*_*p*_^2^ = 0.002, and no interaction, *F*(1, 191) = 0.07, *p* = 0.793, *η*_*p*_^2^ = 0.000. Our findings therefore conceptually replicated the interpolated-testing benefits reported by Jing et al. (2016, Study 2) and Szpunar, Khan, et al. (2013).

To contextualize the interpolated-testing effect size on TUTs, we conducted a *t*-test comparing testing and restudy groups (collapsed across pretest-match conditions); Table 2 indicates a corresponding BF that does not provide supporting evidence that the data were more likely under either the alternative or the null hypothesis, along with a conventionally small-to-medium effect size (Cohen’s *d* =  − 0.29).

As further perspective on effect size (see Magnusson, 2020), the Cohen’s *d* of − 0.29 corresponds to: (a) 61.4% of the restudy group having a higher TUT rate than the mean TUT rate for the testing group (Cohen’s U_3_), (b) an 88.5% overlap between the TUT-rate distributions for the restudy and testing groups, and (c) a 58.1% chance that a randomly chosen subject from the restudy group would have a higher TUT rate than a randomly chosen subject from the testing group. Thus, although we replicated a significant testing effect on TUT rate, it was modest in magnitude and not compelling from a Bayesian perspective.

As an exploratory follow-up analysis, we examined the time-course of mind wandering across the video lecture, to see (a) whether a stronger interpolated-testing effect might be evident later in the lecture, where TUT rates typically rise (as they did in Kane et al., 2017), or (b) whether a content-matched pretest effect might be evident only in early blocks, closest to the pretesting experience (where memory for pretested topics should be best). To do so, for each subject, we calculated a TUT rate for each of the 5 blocks and entered the values into a 2 (Interpolated Activity) × 2 (Pretest-Content Match) × 5 (Video Block) mixed ANOVA, with video block as a repeated measure. The ANOVA (conducted using the Greenhouse–Geisser correction for sphericity to account for within-subject manipulations) indicated a main effect of block, *F*(3.66, 698.32) = 18.85, *p* < 0.001, η_p_^2^ = 0.090, but no significant interactions involving testing or pretesting. Our experimental manipulations did not appear to affect TUT-rate trajectories across the lecture.

**Fig. 4:**
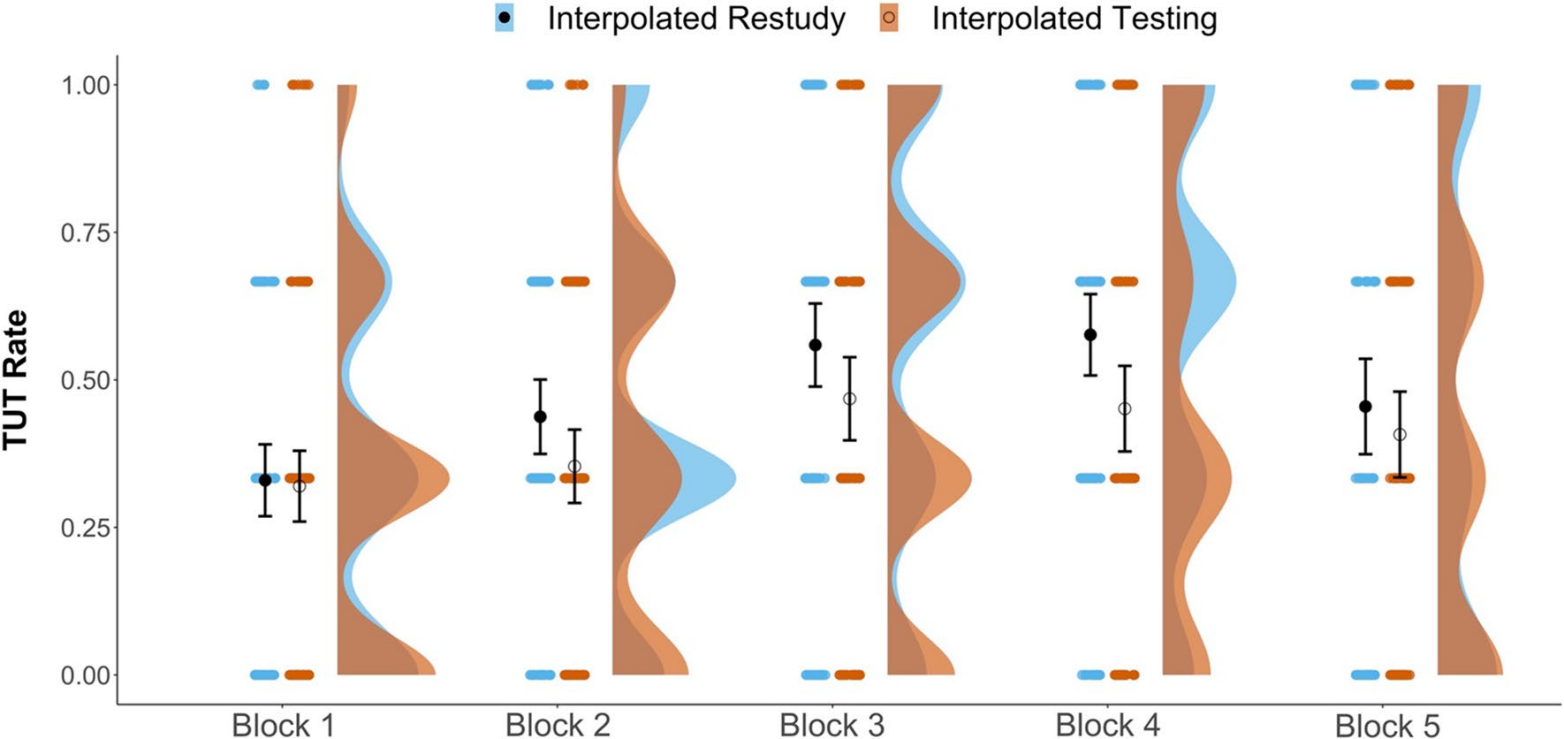
Raincloud plots depicting TUT rates (the proportions of thought reports indicating TUTs) by block in each interpolated activity condition. Dots represent individual subjects’ TUT rates. The closed black dots represent group-level mean estimates for the Restudy conditions; open circles represent the group-level mean estimates for the Testing conditions. Error bars are 95% confidence intervals

Although no significant interaction with interpolated activity was indicated, we note that Fig. 4 shows no evidence of an interpolated-testing effect on TUTs in Block 1, before any test was presented. As would be expected if interpolated tests exerted a causal effect on mind wandering, TUT rates diverged between the testing and restudy groups only after the first interpolated test following Block 1. We therefore conducted an additional exploratory analysis to see whether we (and prior studies) underestimated the effect of interpolated testing on TUTs by including TUT rates from the first part of the video lecture, before any testing had occurred.

Here, we recalculated each subject’s overall TUT rate by including thought-probe responses from only Blocks 2–5 and used these as the dependent measure in a 2 (interpolated activity) × 2 (pretest-content match) ANOVA. Of most importance here, the effect of interpolated activity was again significant, *F*(1, 91) = 5.31, *p* = 0.023, *η*_*p*_^2^ = 0.027, with lower TUT rates for subjects in the interpolated-testing condition (*M* = 0.42) than in the restudy condition (*M* = 0.51). The difference between groups was numerically somewhat larger, and the *p*-value somewhat smaller, than in our original analysis, but the effect-size estimates were similar.

Indeed, a *t*-test comparing testing and restudy groups (collapsed across pretest-match conditions) yielded BF = 1.78, indicating only anecdotal evidence that the data were more likely under the alternative than the null hypothesis. It also indicated a Cohen’s *d* =  − 0.33 [− 0.61, − 0.05], which closely matches our originally calculated effect size (*d* =  − 0.29) and is still considerably smaller than those reported in prior studies (Jing et al., 2016; Szpunar, Khan, et al., 2013). In conclusion, then, we did not greatly underestimate the effect of interpolated testing on TUT rates by including Block 1 thought probes that occurred before the first interpolated test.

#### Rates of other off-task thought reports

**Fig. 5:**
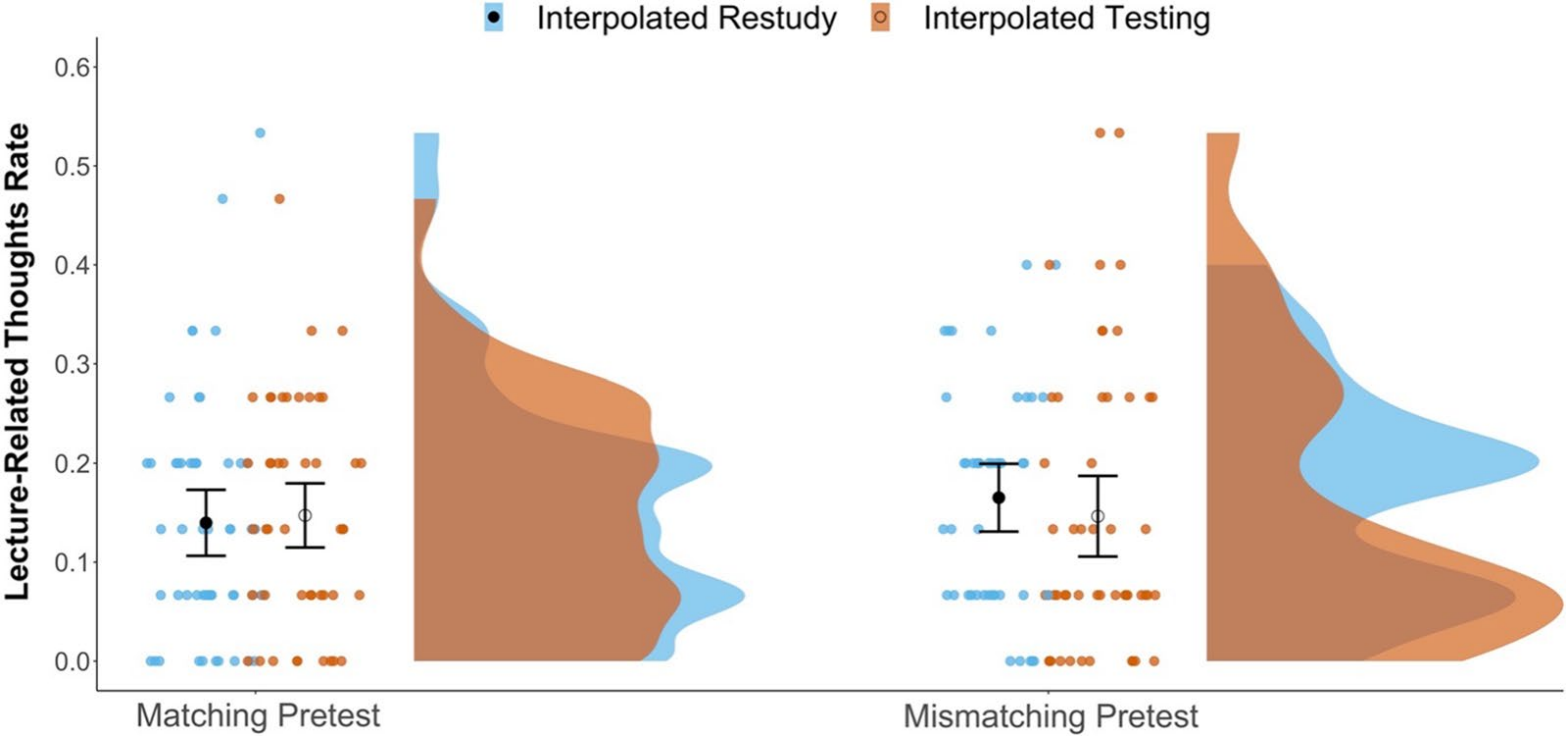
Raincloud plots depicting the proportions of lecture-related off-task thought in each condition. Dots represent individual subjects’ rates. The closed black dots represent group-level mean estimates for the Restudy conditions; open circles represent the group-level mean estimates for the Testing conditions. Error bars are 95% confidence intervals

We next examined whether interpolated activity or pretest-content matching affected rates of reported lecture-related off-task thoughts. As illustrated in Fig. 5 (see also Table 1), the 2 × 2 ANOVA on lecture-related off-task thoughts indicated no significant effects of interpolated activity, *F*(1, 191) = 0.10, *p* = 0.751, *η*_*p*_^2^ = 0.000, or pretest-content match, *F*(1, 191) = 0.48, *p* = 0.489, *η*_*p*_^2^ = 0.003, and no interaction, *F*(1, 191) = 0.55, *p* = 0.460, *η*_*p*_^2^ = 0.003. Table 2 also shows BFs indicating modest evidence that the data were more likely under the null than the alternative model for both the interpolated-testing effect and the pretest-matching effect. We therefore failed to conceptually replicate the significant interpolating-testing effect on lecture-related off-task thoughts reported by Jing et al., (2016, Experiment 2), where *M* report rates were approximately 0.20 and 0.10 for interpolated testing and restudy groups, respectively.

We also conducted a 2 × 2 ANOVA on comprehension-related thoughts (see Table 1). It indicated no significant effects of interpolated activity, *F*(1, 191) = 0.43, *p* = 0.515, η_p_^2^ = 0.002, pretest-content match, *F*(1, 191) = 1.21, *p* = 0.274, *η*_*p*_^2^ = 0.006, or their interaction, *F*(1, 191) = 0.35, *p* = 0.556, *η*_*p*_^2^ = 0.002; Table 2 presents BFs indicating modest evidence that the data were more likely under the null model than the alternative model for the effects of both interpolated activity and pretest-content match.

### Secondary analysis of posttest performance

**Fig. 6:**
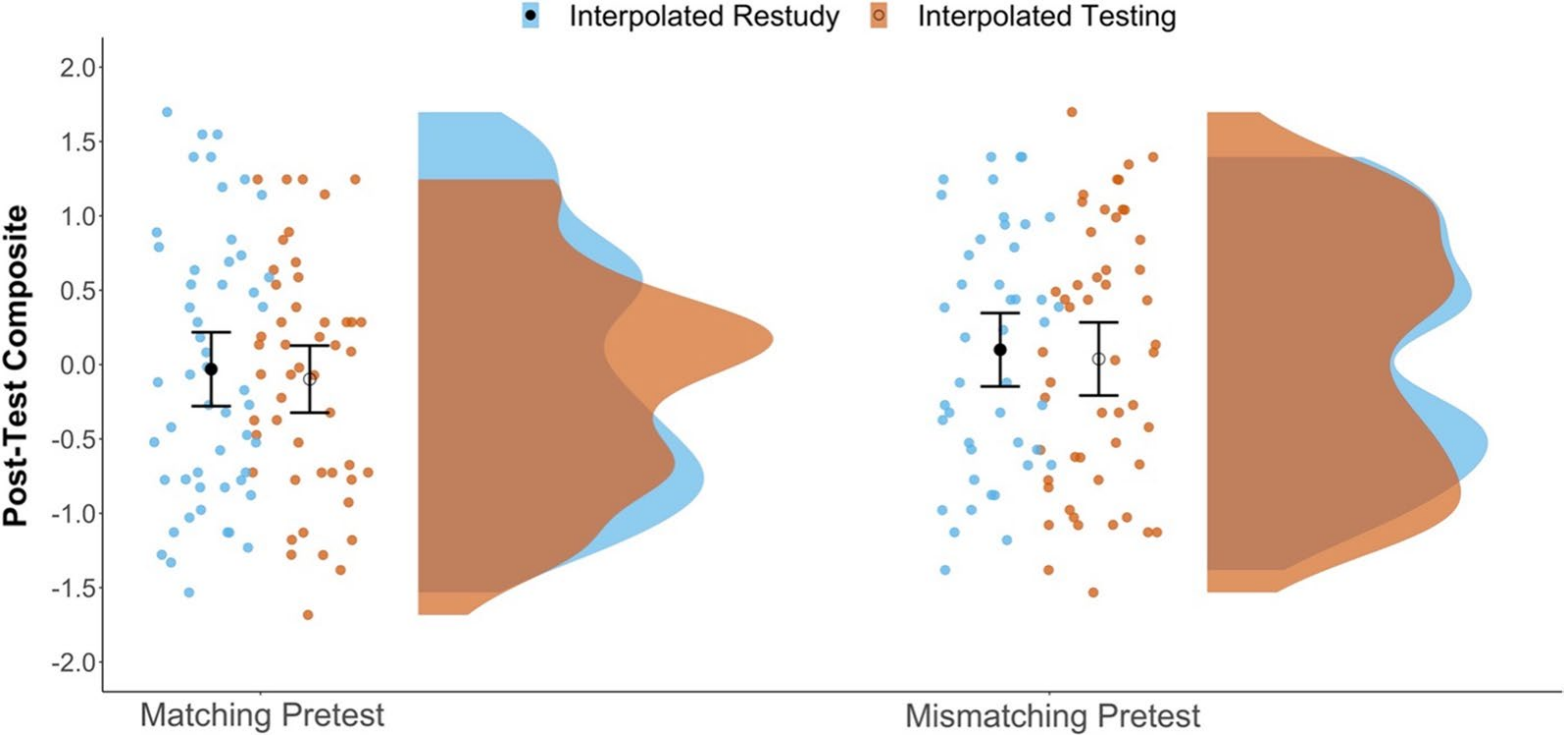
Raincloud plots depicting differences in posttest performance between conditions. Dots represent individual subject means in each condition. The closed black dots represent group-level mean estimates for the Restudy conditions; open circles represent the group-level mean estimates for the Testing conditions. Error bars are 95% confidence intervals

Figure 6 presents the posttest data. A 2 × 2 ANOVA did not indicate a main effect of interpolated activity (i.e., no interpolated-testing effect on posttest performance), *F*(1, 191) = 0.28, *p* = 0.595, *η*_*p*_^2^ = 0.001, or of pretest-content match, *F*(1, 191) = 1.22, *p* = 0.271, η_p_^2^ = 0.006, or an interaction between the two, *F*(1, 191) = 0.00, *p* = 0.982, *η*_*p*_^2^ = 0.000. As seen in Table 2, the BFs for the difference between interpolated testing and restudy groups indicated modest-to-strong evidence that the data were more likely under the null than the alternative model. In short, we did not find conventional benefits for either interpolated testing or content-matched pretesting on final test performance.^2^

As an additional way to assess a possible testing effect in our posttest data, we examined whether subjects in the interpolated-testing condition improved more from pretest to posttest than did subjects in the interpolated-restudy condition. To do this, we selected all subjects in the matched-pretest conditions (*n* = 100) and compared pretest scores to Part 1 of the posttest, which presented the identical 10 multiple-choice items. Scores increased significantly from pretest to posttest, indicating that subjects learned from the lecture, *F*(1, 98) = 84.14, *p* < 0.001, *η*_*p*_^2^ = 0.462. We did not find, however, a significant interpolated activity (testing vs. restudy) × pretest-to-posttest interaction, *F*(1, 98) = 0.98, *p* = 0.325, *η*_*p*_^2^ = 0.010, again providing no evidence for test-potentiated learning (i.e., no benefit of interpolated testing over restudy for subsequent learning).

In Appendix B, we explore the possibility that performance levels on the interpolated tests affected the results here. Specifically, we asked whether interpolated testing produced limited benefits in TUT reduction or learning because subjects did not perform well enough on the interpolated tests. The findings are ambiguous, but we report them for archival purposes.

### Secondary analysis of situational interest

**Fig. 7:**
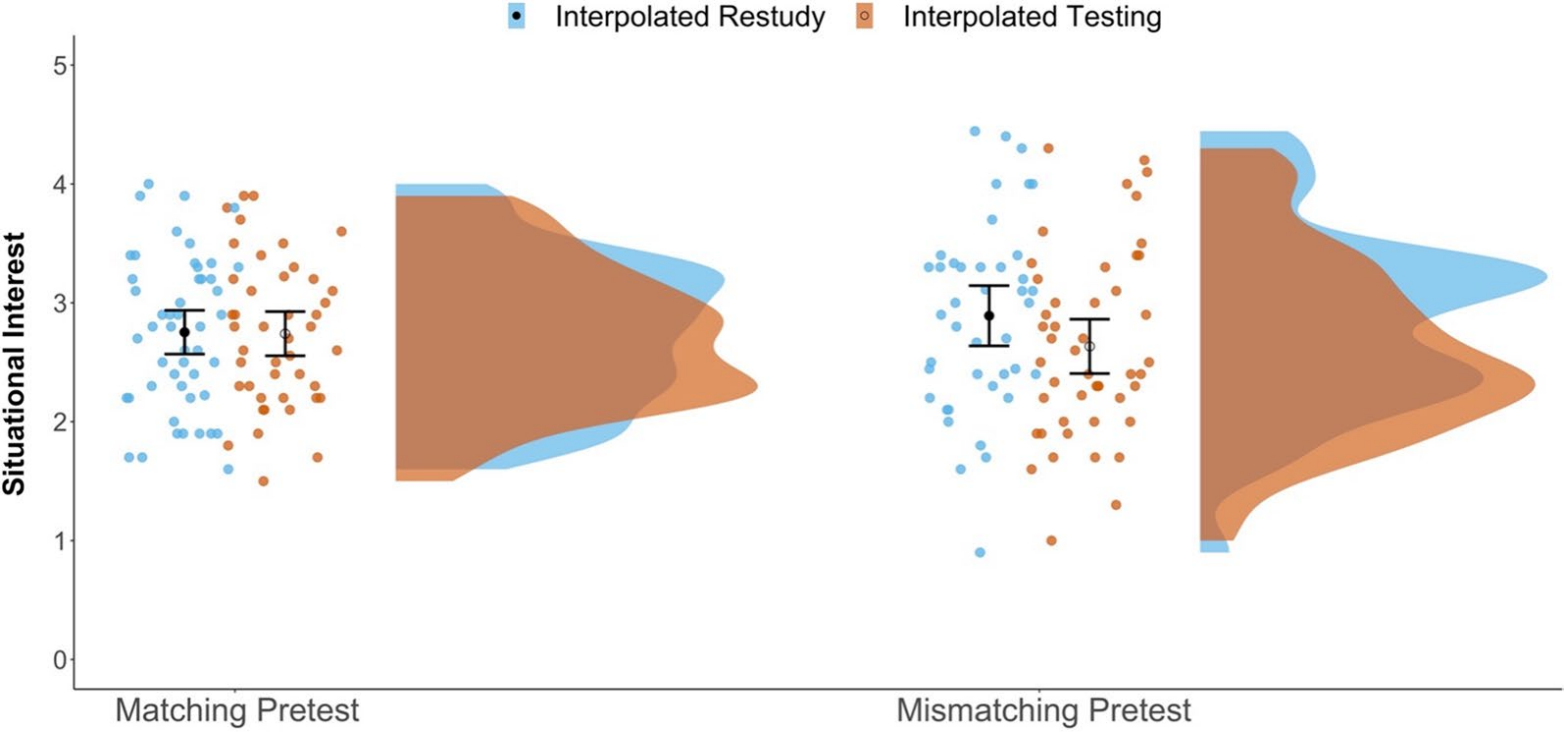
Raincloud plots depicting differences in situational interest ratings between conditions. Dots represent individual subject means in each condition. The closed black dots represent group-level mean estimates for the Restudy conditions; open circles represent the group-level mean estimates for the Testing conditions. Error bars are 95% confidence intervals

Following Jing et al. (2016), here we tested whether interpolated activity or pretest-content matching affected self-reported post-video situational interest in the lecture or the broad topic of statistics (as noted previously, we dropped data from 14 subjects who failed the embedded attention check). As suggested by Fig. 7 (see also Table 1), the 2 × 2 ANOVA indicated no effect of interpolated activity (i.e., no interpolated-testing effect), *F*(1, 177) = 1.62, *p* = 0.204, *η*_*p*_^2^ = 0.009, consistent with findings from Jing et al. (2016), or of pretest content match, *F*(1, 177) = 0.02, *p* = 0.881, *η*_*p*_^2^ = 0.000, and no interaction between the two, *F*(1, 177) = 1.34, *p* = 0.249, *η*_*p*_^2^ = 0.008. Table 2 presents BFs indicating modest-to-strong evidence for the data being more likely under the null than the alternative model for the effects of interpolated activity and pretest content matching.

### Exploratory correlational analyses

Our goal for these analyses was to assess the replication of previously reported significant associations between off-task thought types (i.e., TUT and lecture-related) and outcomes (i.e., posttest performance and situational interest in the lecture) using these video-lecture and assessment materials (Kane et al., 2017). We approached these analyses in two ways: (a) using the whole sample, collapsed across all manipulations, and (b) separately assessing correlations within the testing and restudy groups while collapsing across pretest-match conditions. We consider these analyses not only secondary but also “exploratory”—and we interpret them cautiously—because in both cases we collapsed over conditions that may have affected individual differences without demonstrating robust experimental effects, and in the latter cases our samples were too small to allow precise estimates of correlational effect sizes (Schönbrodt & Perugini, 2013).

Table 3 presents the relevant correlations from the present study and from Kane et al. (2017). Although the correlations from Kane et al. (2017) were generally stronger than those found here, the present *r* values from the full sample were all within 0.06–0.11 of the Kane et al. (2017) values (and all within the originals’ 95% confidence intervals). The correlations from the separate interpolated-testing and restudy groups were more variable, and some were not significant, but that is not surprising given their smaller samples sizes. We thus conclude that the present study replicated the primary correlational results from Kane et al. (2017): (a) the strong negative correlations between TUT rates and posttest performance and situational interest and; (b) the modest positive correlations between lecture-related off-task thoughts and posttest performance and situational interest.^3^

**Table 3:** Correlations between off-task thought types and other variables, in the present study (both for the full sample and separately for each interpolated-activity condition, i.e., testing versus restudy) and in the methodologically similar Kane et al. (2017) study

Off-task thought	Correlate	Study/sample	Correlation [with 95% CI]
TUT	Posttest composite	Present/full	*r*(193) = − .39 [− .50, − .26]*
		Present/testing	*r*(97) = − .40 [− .54, − .22]*
		Present/restudy	*r*(94) = − .40 [− .55, − .21]*
		Kane et al. (2017)	*r*(180) = − .48 [− .58, − .36]*
	Situational interest	Present/full	*r*(179) = − .47 [− .58, − .35]*
		Present/testing	*r*(89) = − .41 [− .57, − .22]*
		Present/restudy	*r*(88) = − .57 [− .70, − .41]*
		Kane et al. (2017)	*r*(180) = − .56 [− .65, − .45]*
Lecture-related	Posttest composite	Present/full	*r*(193) = .15 [.01, .29]*
		Present/testing	*r*(97) = .13 [− .07, .32]
		Present/restudy	*r*(94) = .18 [− .03, .36]
		Kane et al. (2017)	*r*(180) = .26 [.12, .39]*
	Situational interest	Present/full	*r*(179) = .20 [.06, .34]*
		Present/testing	*r*(89) = .04 [− .16, .25]
		Present/restudy	*r*(88) = .36 [.17, .53]*
		Kane et al. (2017)	*r*(180) = .26 [.12, .39]*

*p < .05

## Discussion

Educationally relevant research (and its application to the classroom) has recently broadened its focus beyond memory and metacognition to pay more attention to failures of attention (for a popular review, see Lang, 2020), and particularly to mind wandering (e.g., Risko et al., 2011; Smallwood et al., 2007; Szpunar, Moulton, et al., 2013; Unsworth et al., 2012). Ample evidence from video and live lectures, in laboratory and classroom contexts, shows that TUTs during learning predict disruptions to encoding and comprehension (e.g., Hollis & Was, 2016; Kane, Carruth, et al., 2021; Kane et al., 2017; Risko et al., 2013; Varao-Sousa & Kingstone, 2015; Wammes et al., 2016a, b; Wammes & Smilek 2017). Indeed, with our lengthy (~ 50 min) video lecture on statistics, we replicated prior findings of frequent mind wandering during video lectures (*M* TUT rate ≈ 0.40–0.50) and a negative correlation between TUT rate and posttest test performance (*r* ≈ − 0.40); these replicated findings include the key correlations reported by our previous study using these same learning materials (Kane et al., 2017).

The present laboratory study drew upon a smaller literature on behavioral interventions, such as interpolated testing and pretesting, that might reduce TUTs in learning contexts (Jing et al., 2016; Pan et al., 2020; Szpunar, Khan, et al., 2013). If interpolated testing or pretesting reduce TUTs, it not only presents a practical solution to an applied educational problem, but also suggests that basic theoretical work might profitably expand to consider how attentional mechanisms contribute to testing and pretest effects in learning and memory (e.g., Kornell & Vaughn, 2016; Metcalfe, 2017; Pan & Rickard, 2018), especially in ecologically valid contexts where subsequent learning builds on prior learning (e.g., Chan et al., 2018).

We designed the present study to address concerns regarding sample sizes, measurement limitations, and potential confounds (e.g., effects of notetaking) in prior work in this area (Jing et al., 2016; Pan et al., 2020; Szpunar, Khan, et al., 2013). The study was well powered to detect medium-sized main effects (of interpolated testing and content-matched pretesting). It used well validated thought probes to assess TUTs (and lecture-related off-task thought), and it prevented notetaking to clarify the mechanisms of any potential testing or pretesting benefits. It also contrasted a matched-pretest group to a mismatched-pretest group, to isolate the possible mechanism of any pretesting effect on TUTs found here (i.e., scaffolding attention to the foreshadowed, critical topics).

### Interpolated testing and TUT rate

We conceptually replicated prior findings (Jing et al., 2016; Szpunar, Khan, et al., 2013) that students given periodic tests within a lecture reported significantly fewer TUTs (*M* rate = 0.40) than did those who restudied the same information (*M* rate = 0.47); we also replicated the Jing et al. (2016) finding that interpolated testing did not increase situational interest in the lecture, despite reducing TUTs. Consistent with the metacognition framework for explaining test-potentiated new learning (see Chan et al., 2018), the interpolated-testing benefit over restudying suggests that testing works by providing students with feedback on their learning from the prior portions of the lecture, which then motivates greater attention.

The collective results across studies, however, suggest that this interpolated-testing effect on TUT rate yields highly variable standardized effect sizes: Two prior experiments reported Cohen’s *d*s of approximately 1.0 (Jing et al., 2016, Experiment 2, *n* = 18 per group; Szpunar, Khan, et al., 2013, *n* = 16 per group), one prior experiment reported a nonsignificant testing effect (*d* = 0.15; Jing et al., 2016, Experiment 1, *n* = 18 per group), and the present study reported a just-significant effect with a modest *d* = 0.29 (*n* = 96–100 per group). Moreover, the BF for the present study’s effect of interpolated testing on TUTs indicated anecdotal evidence supporting the *null* hypothesis.

Some of this effect-size variability across studies is likely due to small sample sizes, which produce noisy effect-size estimates (e.g., Perugini et al., 2014; Schäfer & Schwarz, 2019). As well, standardized effect sizes are products not only of intervention strength but also of the entire study design, including heterogeneity within the studied sample (e.g., Simpson, 2018). It is possible, then, that the larger effect sizes from prior studies arose from testing only Harvard University students (Szpunar, Khan, et al., 2013) or an unspecified mix of Harvard and Boston University students (Jing et al., 2016). Both samples were likely much more intellectually homogeneous than students at a comprehensive state university, such as UNCG, which should reduce the ratio of noise to signal and thus produce larger effect sizes.

With that said, any generalizations from this small literature are challenging for many reasons: These few studies are so methodologically diverse that effect sizes might vary systematically with aspects of the study design, such as subject sample, video topic and length, number of thought probes, thought-probe format, number of interpolated tests and their format, interpolated-test difficulty, allowing or not allowing notetaking, posttest retention interval and difficulty, and extent of subjects’ prior knowledge on the lecture topic. Future research on the effect of interpolated testing on TUTs should thus take designing-for-variation and meta-analytic approaches to estimating effect size and its robustness (e.g., Baribault et al., 2018; Brunswik, 1955; Fyfe et al., 2021; Greenwald et al., 1986; Harder, 2020; Landy et al., 2020).

We were able to provisionally rule out one possible explanation for the small effect of interpolated testing on TUTs found here, however. Our lecture video was longer (52 min) than those used in prior studies (21 min in Szpunar, Khan, et al., 2013; 40 min in Jing et al., 2016), and most learning studies find that TUT rates increase substantially over the lecture period (e.g., Cohen et al., 1956; Kane, Carruth, et al., 2021; Kane et al., 2017; Lindquist & McLean, 2011). Perhaps, then, we underestimated the testing benefit on TUTs because the negative effects of time-on-task were stronger than the benefits of interpolated testing. Although we replicated prior findings of TUT rates increasing over the lecture here, we did not find an interaction of lecture block with interpolated activity. TUT rates increased similarly for the testing and restudy groups across the lecture, with no sign of an early benefit of interpolated testing over restudy that diminished with time.

As a final interpretive point, we consider here that the present study produced a small but significant interpolated-testing effect on TUTs but no significant testing effect on subsequent posttest performance, unlike prior studies (Jing et al., 2016; Szpunar, Khan, et al., 2013). This null posttest finding may appear peculiar on the surface, given that learning and TUT experiences are likely linked. One major difference between our study and the prior studies, however, is that our subjects were not allowed to take notes during the lecture. It is thus possible that notetaking—which increased significantly under interpolated testing—contributed to these prior findings of interpolated-testing effects on posttest performance (Jing et al., 2016; Szpunar, Khan, et al., 2013).

More generally, other aspects of our study design may have minimized the size of the interpolated testing effect on memory (i.e., on the lecture posttest), based on moderator results from recent meta-analyses (Adesope et al., 2017; Rowland, 2014). For example, our posttest contained recognition and free-response items, which produce weaker testing effects on memory than does cued recall (*g* = 0.29 vs. 0.61; Rowland, 2014).^4^ Further, we had a brief retention interval, which reduces testing effects on memory relative to longer retention intervals (*g* = 0.56 vs. 0.82, Adesope et al., 2017; *g* = 0.41 vs. 0.69; Rowland, 2014). As well, our interpolated tests and the final test presented different items (sometimes on different lecture subtopics), which reduces testing effect sizes relative to matching items (*g* = 0.53 vs. 0.63; Adesope et al., 2017). Finally, we did not provide feedback about initial learning or following the interpolated tests, which one meta-analysis (Rowland, 2014) found to reduce testing effects on memory (no feedback: *g* = 0.39 vs. feedback: *g* = 0.73; but see Adesope et al., 2017, with *g*s = 0.60 vs. 0.63, respectively).^5^

We might have found a larger testing effect on posttest recall if we had used a longer retention interval, if we had matched posttest items to interpolated-test items, or if we had focused our posttest on cued-recall items. None of these variables, however, could have retroactively affected mind wandering that had already occurred during the lecture. That is, because several mechanisms contribute to interpolated testing effects on final recall but not on in-lecture TUTs, and because any interpolated-testing effects on TUTs should have some downstream consequences for learning—rather than vice versa—the finding of large, small, or null testing effects on final memory tests should not be considered diagnostic for evaluating the evidence for interpolated-testing effects on TUTs.

### Content-matched pretesting and TUT rate

Building on Pan et al. (2020), who found that pretesting, either before each video segment or before the entire video, reduced retrospective TUT ratings relative to no-pretest controls (*d*s = 0.39, 0.74, and 0.91), we found no effect of content-matched pretesting versus content-mismatched pretesting in reducing TUT reports to in-the-moment thought probes (*M* TUT rates = .43 and .44 for matching and mismatching pretest groups, respectively; *F* < 1). Although these conflicting results may reflect sampling error, they were likely driven by the different control conditions across studies.

Whereas Pan et al. (2020) compared pretested subjects to those who completed an unrelated task (algebra problems), as is typical of the pretesting literature, we compared pretested subjects to those who also completed a pretest on lecture-*related* topics not appearing in the video or posttest. So, here, we found that subjects provided with advance warning of the topics to be covered in (and tested from) the lecture did not mind-wander less than did subjects who were uninformed about the *specific* topics to be covered in (and tested from) the lecture.

If our null content-matched pretesting findings are replicable, they suggest that any pretesting benefit on TUTs does not arise from highlighting to subjects what specific information they should most closely attend to during the lecture. Such pretesting benefits, such as that reported by Pan et al. (2020), might instead arise from the more general feedback that subjects receive from completing a challenging pretest that demonstrates their lack of knowledge. Although, as noted earlier, effects of pretesting *on memory* may sometimes be limited to material that matches what was included in the pretest (e.g., Pressley et al., 1990; Richland et al., 2009), any effects of pretesting (versus no pretesting) *on TUTs* may be due to pretesting increasing curiosity or the motivation to attend and reduce the knowledge deficit, that is, by the same mechanism that is likely responsible for any interpolated-testing effect on TUTs.^6^

The lack of a pretesting-content match on learning in the present study might be attributable to subjects’ failure to remember the pretest items (or topics) during the lecture. That is, subjects who took the matched pretest might not have processed the items deeply enough to remember them (and any errors they made on those items) while watching the video or while taking the posttest. For example, St. Hilaire and Carpenter (2020) reported a pretesting benefit for learning only in cases where subjects remembered the pretested items during the video lecture. Given that our video lecture was so lengthy (see Geller et al., 2017)—at over twice the duration of the Pan et al. (2020) lecture—and that our pretest material was unfamiliar to most subjects, they may have not been able to effectively associate the ongoing lecture with the pretest.

### Interpolated testing, content-matched pretesting, and lecture-related off-task thought

Students in both classroom and laboratory studies sometimes report thoughts that are not about the here-and-now of a lecture but that are nonetheless conceptually related to the topic (e.g., thinking about earlier lecture points, or connecting lecture material to everyday life; Locke & Jensen, 1974; Schoen, 1970; Shukor, 2005). Such lecture-related mind-wandering might even be helpful (perhaps akin to elaboration effects in memory; Craik & Tulving, 1975), as it correlates positively with learning from that lecture (Jing et al., 2016; Kane et al., 2017).

The present study replicated the modest positive correlation between lecture-related off-task thought and posttest performance (*r* = 0.15). However, whereas Jing et al. (2016) reported that interpolated testing both decreased TUTs and increased lecture-related off-task thoughts during a video lecture, we did not find either interpolated testing or the match in pretesting content to increase lecture-related off-task thoughts in a larger subject sample. Again, a designing-for-variation approach in future work, with well-powered studies and meta-analyses, might indicate the dependency of any association between lecture-related off-task thought and learning to particular aspects of the learning or testing context.

### Additional limitations and constraints on generality

While the present study arguably has some strengths compared to prior studies of testing and pretesting on TUTs, there are limitations worth noting that we have not yet discussed. First, like most studies of TUTs in educational contexts, the present investigation was limited in using a convenience sample of North American undergraduates from a single university (albeit a university with a relatively diverse student body).

Second, our randomization process did not yield sufficiently similar groups across testing conditions after exclusions, as subjects in our interpolated-restudy groups scored higher on the pretest, on average, than did subjects in our interpolated-testing groups. Although we conducted all analyses both with and without pretest scores as a covariate, and although pretest scores did not significantly predict any of our thought-report outcomes, confidence in our conclusions would be stronger had our design produced better-matched groups.

Third, like most investigations of TUTs during video lectures, our study design did not match some aspects of university students’ real-world learning from video materials. Subjects were not able to pause or rewind the lengthy video, to take notes, or to ask questions about the lecture content. In controlling the flow of learning material and limiting typical learning aids (which was important to determining whether interpolated-testing effects on TUTs were independent of interpolated-testing effects on notetaking), we may have hampered subjects’ efforts to build integrative mental models of the material. This may then have artificially inflated their tendency for TUTs and the disruptive influence of TUTs on learning.

Finally, some of the current study’s results may have been biased by the thought probes we used—content-focused probes that assessed not only TUTs but also lecture-related and comprehension-related thoughts. Prior work has found reports of these thought types with open-ended probes (Jordano, 2018; Locke & Jensen, 1974; Schoen, 1970), suggesting that they are not always produced as a demand effect. Nonetheless, studies that repeatedly present probes asking about lecture- and comprehension-related off-task thoughts have the potential to bias subjects’ experiences or reporting. If stronger students particularly believe they should have these kinds of thoughts, or that such thoughts are likely to be helpful, they may come to have these thoughts more frequently or simply endorse them more frequently as a socially desirable response. Such selective biasing may contribute to the positive correlation between lecture-related off-task thoughts and learning (see also Jing et al., 2016; Kane et al., 2017), although they cannot explain the lack of correlation between comprehension-related off-task thoughts and learning (see also Kane et al., 2017).

## Conclusion

Consistent with a small number of studies measuring TUTs during video lectures (Jing et al., 2016; Szpunar, Khan, et al., 2013), we found that interpolated tests significantly reduced TUT rates relative to interpolated restudy opportunities, but the standardized effect size was small—considerably smaller than in most prior studies—and the associated Bayes factor suggested inadequate evidence for either the null or the alternative model. The benefits of interpolated testing to engaging students’ attention may thus be smaller or more fragile than anticipated. Indeed, they may be too small or fragile to be of much practical use in reducing TUTs in authentic educational settings.

We did not find that the match in content of a pretest about the upcoming lecture material reduced TUTs compared to a mismatch in content. If the pretesting effect on TUTs found by Pan et al. (2020) is genuine and generalizable, then pretesting may reduce TUTs by showing students how little they know about a general topic and thus motivating them to pay attention (detectable with the Pan et al. design), rather than by highlighting or foreshadowing test-specific material for enhanced attentional focus (detectable with our design).

## Data Availability

All data and materials created for the present study are available at the OSF site, https://osf.io/6ujsg/. Video lecture materials used here are available from the Kane et al. (2017) OSF site, https://osf.io/u5bnw/

## Appendix A: Follow-up ANCOVA results for the ANOVA effects of interest

For all the dependent variables below, we re-conducted the reported 2 × 2 ANOVAs as ANCOVAs that controlled for pretest score (standardized within pretest type).

1. *TUT rates.* Of most importance, the ANCOVA indicated a significant effect of interpolated activity (testing versus restudy) on TUT rate, *F*(1, 190) = 4.88, *p* = 0.028, η_p_^2^ = 0.025, as did the ANOVA; the pretest covariate did not predict TUT rate, *F*(1, 190) = 2.01, *p* = 0.158, *η*_*p*_^2^ = 0.010. Also as in the ANOVA, there was no significant effect of pretest-content match, *F*(1, 190) = 0.42, *p* = 0.518, *η*_*p*_^2^ = 0.002, nor an interaction of interpolated activity and pretest-content match, *F*(1, 190) = 0.10, *p* = 0.750, *η*_*p*_^2^ < 0.001.
2. *Lecture-related off-task thought rates.* Pretest scores did not significantly predict lecture-related mind-wandering rates, *F*(1, 190) = 1.68, *p* = 0.197, *η*_*p*_^2^ = 0.009; and, as in the ANOVA, there was no effect indicated for interpolated activity, *F*(1, 190) = 0.01, *p* = 0.906, *η*_*p*_^2^ = 0.000, pretest-content match, *F*(1, 190) = 0.47, *p* = 0.495, *η*_*p*_^2^ = 0.002, or their interaction, *F*(1, 190) = 0.63, *p* = 0.429, *η*_*p*_^2^ = 0.003.
3. *Comprehension-related off-task thought rates.* Pretest scores did not significantly predict comprehension-related thought rates, *F*(1, 190) = 0.62, *p* = 0.431, *η*_*p*_^2^ = 0.003. As in the ANOVA, there was no significant effect of interpolated activity, *F*(1, 190) = 0.28, *p* = 0.600, *η*_*p*_^2^ = 0.001, pretest-content match, *F*(1, 190) = 1.22, *p* = 0.271, *η*_*p*_^2^ = 0.006, or their interaction, *F*(1, 190) = 0.31, *p* = 0.578, *η*_*p*_^2^ = 0.002.
4. *Posttest scores*. As in the ANOVA, there was no significant effect of interpolated activity (i.e., no testing effect), *F*(1,190) = 1.23, *p* = 0.270, *η*_*p*_^2^ = 0.006, or of pretest-content match (i.e., no pretesting effect), *F*(1,190) = 0.00, *p* = 0.995, *η*_*p*_^2^ = 0.000, and no interaction, *F*(1,190) = 0.01, *p* = 0.907, *η*_*p*_^2^ = 0.000. The pretest score covariate did, however, significantly predict posttest score, *F*(1,190) = 12.48, *p* < 0.001, *η*_*p*_^2^ = 0.062.
5. *Situational interest*. Pretest scores did not significantly predict situational interest, *F*(1, 176) = 2.78, *p* = 0.097, *η*_*p*_^2^ = 0.016. As in the ANOVA, there was no significant effect of interpolated activity, *F*(1, 176) = 1.05, *p* = 0.306, *η*_*p*_^2^ = 0.006, pretest-content match, *F*(1, 176) = 0.02, *p* = 0.900, *η*_*p*_^2^ = 0.000, or their interaction, *F*(1, 176) = 1.52, *p* = 0.219, *η*_*p*_^2^ = 0.009.

## Appendix B: Exploratory analyses of performance on the interpolated tests

The lack of a traditional interpolated-testing effect on learning, and the significant-but-modest interpolated-testing effect on TUTs, suggests that we should consider subjects’ performance on the interpolated tests. Perhaps interpolated testing did not greatly help students to sustain attention or learn because the tests were too difficult to motivate continued or improved effort. For similar reasons, perhaps testing only selectively helped the students who answered more of the interpolated-test items correctly. We addressed these possibilities by analyzing interpolated-test performance (max score = 6 points per interpolated test) from the two interpolated-testing conditions, collapsed across pretest-match conditions (*n* = 98), and assessing correlations between interpolated-test performance and TUT rates and posttest scores.

First, mean points per interpolated test was 2.35 with SD = 0.79. Although these scores were not at floor, they do indicate that subjects typically answered more than half of interpolated-test items incorrectly. The Chan et al. (2018) meta-analysis of test-potentiated new learning indicated, however, that performance levels on interpolated tests did not significantly moderate the effect. These meta-analytic findings suggest that the somewhat low mean performance in our sample is not driving the small interpolated-testing benefits we found here.

Second, interpolated-test performance correlated with our outcome measures of interest. Specifically, interpolated-test performance was significantly correlated with TUT rate, *r*(97) =  − 0.26 [− 0.44, − 0.07], *p* < 0.05, and posttest performance, *r*(97) = 0.68 [0.56, 0.78], *p* < 0.001. Subjects who were more successful in their retrieval practice demonstrated fewer TUTs during the lecture and better mastery of the lecture material. Unfortunately, these correlational findings are causally ambiguous. They might support the claim that interpolated-test performance reduced TUTs and improved learning, or they might instead indicate that better sustained attention and learning increased subjects’ performance on the interpolated tests.

We attempted to examine this issue further, with respect to TUT rates, by assessing how interpolated-test performance correlated with TUTs during the current (just completed) block and subsequent block (e.g., Block 1 Test × Block 1 TUT Rate vs. Block 1 Test × Block 2 TUT Rate). If interpolated-testing performance selectively predicts subsequent TUT rate, that would suggest that testing reduces mind wandering. However, if interpolated-testing performance selectively predicts the just-completed block’s TUT rate, that would suggest that mind wandering reduces test performance.

Table A1 presents the results of these block analyses. Again, the results are ambiguous. In some cases, interpolated-test performance is significantly negatively correlated with current block TUTs, consistent with TUTs causing poorer interpolated-test performance. In other cases, however, interpolated-test performance correlates with subsequent block TUT rates, consistent with interpolated testing causing a reduction in TUTs. Given this ambiguity—not to mention the possible reciprocal influences of interpolated-testing and TUTs on each other—we cannot confidently interpret the directionality of their association.

**Table A1:** Correlations [with 95% CIs] between interpolated-test performance and TUTs, by current block and subsequent block.

	Current block TUT rate	Subsequent block TUT rate
Interpolated Test 1	*r*(97) = .10 [− .10, .29]	*r*(97) = .25 [.06, .43]*
Interpolated Test 2	*r*(97) = − .27 [− .45, − .08]**	*r*(97) = − .25 [− .43, − .06]*
Interpolated Test 3	*r*(97) = − .13 [− .32, .07]	*r*(97) = − .29 [− .46, − .09]**
Interpolated Test 4	*r*(97) = − .20 [− .39, − .01]*	*r*(97) = − .14 [− .33, .06]
Interpolated Test 5	*r*(97) = − .19 [− .37, .01]	

**p* < .05

***p* < .01

## Footnotes

1 Kane et al. (2017) showed that the pretest items from the matched-content condition were challenging in their sample for students with no background in statistics (i.e., fewer than 2% of subjects answered more than three questions accurately while reporting high confidence/not guessing). We did not analyze confidence reports for the current study.

2 The results of a 2 (pretest condition) × 2 (interpolated activity) ANOVA on just Part 1 of the posttest, which was comprised of the same 10 multiple-choice items as the matched-content pretest, suggested no main effect of pretest match, *F*(1, 191) = 0.61, *p* = .435, η_p_^2^ = .003, no effect of interpolated activity, *F*(1, 191) = 0.01, *p* = .933, η_p_^2^ < .001, and no interaction, *F*(1, 191) = 0.02, *p* = .883, η_p_^2^ < .001. Thus, subjects who took a pretest that perfectly matched the eventual final test did not outperform subjects who completed a content-mismatched pretest.

3 Rates of comprehension-related off-task thoughts did not correlate with posttest scores in the present study, *r*(193) =  − .10 [− .24, .04], *p* = . 166; the corresponding correlation from Kane et al. (2017) was numerically positive but near zero, *r*(180) = .01 [− .14, .16]. The correlation was not significant here in either the testing condition, *r*(97) =  − .10 [− .29, .10], *p* = 0.320, or the restudy condition, *r*(94) =  − .10 [− .29, .11], *p* = .360.

4 The primary meta-analytic test statistic for between group comparisons is Hedge’s *g* which is based on a similar formula as Cohen’s *d*. In fact, these two statistics are nearly identical in sample sizes above 20 (Kline, 2004; Lakens, 2013).

5 We acknowledge that one aspect of our design may have promoted *larger* testing effects on final memory, according to Adesope et al. (2017): Our interpolated tests presented items using multiple formats (multiple-choice and free response) rather than just one format (*g* = .80 vs. .70, respectively).

6 We also acknowledge that other methodological differences between the present study and Pan et al. (2020) might have affected the results, including the thought-probe type, video length, and average pretest performance. Moreover, our claim assumes that the Pan et al. control task (algebra problems) did not somehow *suppress* learning relative to other possible controls.
